# Long-Term Outcomes of Patients with Staple Line Leaks Following Sleeve Gastrectomy

**DOI:** 10.1007/s11695-024-07307-0

**Published:** 2024-05-30

**Authors:** Adam Abu-Abeid, Adi Litmanovich, Subhi Abu-Abeid, Shai Meron Eldar, Guy Lahat, Jonathan Benjamin Yuval

**Affiliations:** 1 Division of General Surgery, Tel Aviv Sourasky Medical Center, Tel Aviv University, 6, Weizman St., 6423906 Tel- Aviv, Israel; 2 Division of General Surgery, Bariatric Unit, Tel Aviv Sourasky Medical Center, affiliated to Sackler Faculty of Medicine, Tel Aviv University, 6, Weizman St., 6423906 Tel- Aviv, Israel; 3 Tel Aviv University, The Faculty of Medical & Health Sciences, Tel- Aviv, Israel

**Keywords:** Bariatric surgery, Severe obesity, Sleeve Gastrectomy, Leaks, Long-term outcomes

## Abstract

**Purpose:**

The long-term outcomes of patients with staple line leaks (SLL) after SG are under-reported. The purpose of this study is to evaluate the long-term outcomes of patients with SLL after SG.

**Materials and Methods:**

A retrospective analysis of a maintained patient registry of patients undergoing SG between January 2012 and December 2020 in a single bariatric center was analyzed and included patients with SLL. Outcomes were compared to a group without SLL.

**Results:**

During this period, 1985 patients underwent SG of which 61 patients (3.1%) developed leak. 26 patients (1.3%) had overt SLL and the rest had organ space infection around the staple line. The mean age and body mass index (BMI) were 39.8 ± 11.3 and 41.5 ± 4.7, respectively. 7 patients (11.4%) underwent concomitant surgery and 12 patients (19.7%) had intraoperative complications. Leak was complicated by Clavien-Dindo ≥ 3 in 31 patients (50.8%). Reoperation rate was 27.8% (*n* = 17). ≥ 2 interventions were required in 26% of patients (*n* = 16). Two patients died during hospitalization due to septic complications. Long-term follow-up of median 121 months was available in 78% of the cohort (*n* = 48). The median total weight loss and BMI were 27% and 30.1 kg/m^2^, respectively. Weight loss outcomes were higher in comparison to patients without SLL. Seven patients (14.6%) underwent SG revision. All associated medical problems improved during follow-up except for gastroesophageal reflux which was found in 50% of cohort.

**Conclusion:**

Long-term outcomes of SG patients with SLL are satisfactory in terms of weight loss, resolution of comorbidities, and requirement for surgical revision.

**Graphical Abstract:**

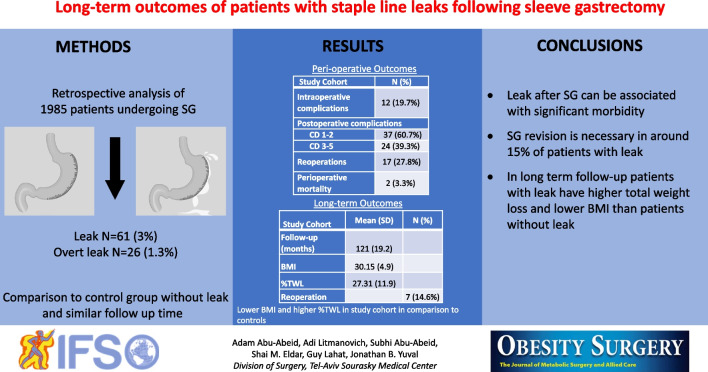

**Supplementary Information:**

The online version contains supplementary material available at 10.1007/s11695-024-07307-0.

## Introduction

Sleeve gastrectomy (SG) is the most common Metabolic and Bariatric Surgery (MBS) with nearly 400,000 SG surgeries performed per year around the world [1]. The advantages of SG, include a high safety profile with low perioperative morbidity and mortality; and good efficacy in terms of substantial and sustained weight loss, resolution of obesity-associated medical problems and improvement in quality of life [2–4].

However, SG is not devoid of complications including severe infectious complications. Overt staple line leaks and occult leaks without contrast extravasation or enteric content in the drain, such as organ space infections around the staple line are dreaded complications of SG. Leaks are reported in 1.2–2.4% of SG procedures, with adverse effects on early outcomes including the need for antibiotic treatment, reoperation, several interventions, increased hospital length of stay [5, 6] and may even lead to mortality in 5–10% of patients with staple line leak [3, 7, 8].

The management of staple line leak is usually tailored individually to the patient depending on the timing of presentation, whether the leak is contained or not, and on patients’ clinical status; and includes intravenous antibiotics, percutaneous drainage, endoscopic stent placement, and reoperation [7].

Furthermore, as in other MBS, SG carries a possible need for revisional surgery in the long run, mainly due to insufficient weight loss, weight regain, and gastroesophageal reflux [9, 10]. To our knowledge, long-term outcomes (of more than a couple years) of patients that have experienced staple line leaks after SG is underreported in the literature.

The purpose of this study was to evaluate perioperative and long-term outcomes of SG patients who experiences staple line leak following SG. It was our hypothesis that these patients would have similar long-term outcomes in terms of weight loss and resolution of comorbidities once they overcame the initial infectious complication.

## Methods

### Patient Data

This is a retrospective study analysis of a prospectively maintained SG patient registry in a single tertiary center (January 1st 2012 to December 31st 2020). All patients were adults (18 years and above) and underwent a multi-disciplinary evaluation for MBS, prior to surgery. All patients who experienced deep surgical site infection were included in the current study. Leak was defined as either overt staple line leak or organ space infection abutting the staple line. The diagnosis of overt staple line leak was confirmed on one or more of the following: imaging studies (i.e., computed tomography, upper gastrointestinal series), enteric discharge in the abdominal drain, or at reoperation.

The following clinical data were captured from our registry. Baseline characteristics including gender, age, body mass index (BMI), previous bariatric procedure, smoking, and obesity related medical problems including type 2 diabetes (T2D), hypertension (HTN), hyperlipidemia (HL), gastroesophageal reflux disease (GERD), obstructive sleep apnea syndrome (OSAS), and non-alcoholic fatty liver disease (NAFLD). Perioperative data included: the American Society of Anesthesiologists (ASA) score [11], concomitant surgery, intraoperative complications, Clavien-Dindo (CD) score [12], reoperation, number of re-interventions, intensive care unit admission, total length of stay (LOS), and mortality. Long-term follow-up included: follow-up time, last follow-up BMI, total weight loss (TWL), chronic complications, reoperations, medical treatment for severe obesity, and death during follow-up.

A control group of patients without staple line leak after SG and similar follow-up was compared to the study cohort.

The study was approved by the Institutional Review Board and was performed in accordance with the ethical standards of the institutional and/or national research committee and with the 1964 Declaration of Helsinki and its later amendments or comparable ethical standards. Informed consent was obtained from all individual participants included in the study.

### Surgical Technique

SG was performed after the administration of venous thromboembolism prophylaxis (subcutaneous injection of heparin 5000 units) and antibiotic prophylaxis (intravenous cephazolin 2–3 g). The abdomen was insufflated through Veress needle with carbon dioxide to a pressure of 15 mmHg, and 5 laparoscopic ports were inserted. The greater curvature of the stomach was mobilized, starting 4 cm proximal to the pylorus up to the Angle of His. A 32-to 36-F bougie was inserted along the lesser curvature, and the stomach was vertically transected with linear staplers, to create the gastric sleeve. Staple line reinforcmenet was not routinly applied. A drain was left along the staple line by decision of operating surgeon.

### Post-operative Course

All patients were admitted to the surgical ward. Liquid diet was resumed on the first post-operative day (POD1). Physical examination was performed daily and blood work was performed on POD1. Upper GI flouroscopy was not performed routinely. Stable patients with clinical suspicion of deep surgical site infection (SSI) underwent fluid resucitation, were given intervenous antibiotics and underwent a CT scan to confirm the diagnosis and guide further interventions. Unstable patients were taken for surgical re-exploratoin.

### Statistical Analysis

Statistical analysis was performed using SPSS version 27.0. Continous variables are presented as mean and standard deviation (SD). Proportions are presented as *n* (%). Differences between groups were evaluated using the Pearson chi-squared test, Wilcoxon rank sum test, or Fisher’s exact test. Two-sided *p* values < 0.05 were considered statistically significant.

## Results

During the study period, 1985 patients underwent SG. The study cohort consists of 61 patients (3.1%) who developed SG-related staple line leaks, and in 26 of them (42.6%) overt staple line leak was confirmed, comprising 1.3% of the total SG procedures performed. The other 35 patients (57.3%) had fluid collection or abscess abutting the staple line without overt demostration of leak.

The baseline characteristics of 61 patients in the study cohort are shown in Table 1—59% of patients were women, mean age was 39.8 + / − 11.3, the mean ASA score was 2.7 + / − 0.5, and the mean BMI was 41.5 + / − 4.7. Comparison of baseline characeteristics of the study group to the control group can be seen in Supplementary Table 1 and showed no meaningful difference between groups. Perioperative data are shown in Table 2, 11.4% (*n* = 7) underwent a concomitant surgery of which 3 patients underwent band removal, 2 patients underwent cholecystectomy, 1 patient underwent umbilical hernia repair and 1 patient underwent a disconnection of a previous Nissen fundoplication. Intraoperative complications were reported in 19.7% (*n* = 12) of patients. In these 12 patients, intraoperative complications included bleeding in 9 (75%), and inadvertent gastrotomy in 3 patients (25%). Thirty-one patients (50.8%) required invasive early reintervention via gastroscopy, invasive radiology, or surgery. Reoperation rate was 27.8% (*n* = 17) and 11 patients were found to have staple line leak intraoperatively. Several (≥ 2) reinterventions during the hospital stay were required in 26% of the study cohort (*n* = 16). The mean LOS was 22.3 days + / − 20.2 for this cohort. In-hospital mortality rate was 3.3% (*n* = 2), both due to septic complications of staple line leak.

**Table 1:** Baseline characteristics of patients with staple line leaks after SG (*n* = 61)

Characteristic	
Age, mean (SD)	39.8 (11.3)
Male gender, *n* (%)	25 (41%)
BMI, mean (SD)	41.5 (4.7)
ASA score, mean (SD)	2.7 (0.46)
T2D, *n* (%)	18 (29.5%)
Hypertension, *n* (%)	27 (44.3%)
Hyperlipidemia, n (%)	19 (31.2%)
OSA, *n* (%)	9 (14.8%)
GERD, *n* (%)	5 (8.2%)
NAFLD, *n* (%)	50 (82%)

*SG*, sleeve gastrectomy; *SD*, standard deviation; *BMI*, body mass index; *ASA*, American Society of Anesthesiologists; *T2D*, type 2 diabetes; *OSA*, obstructive sleep apnea; *GERD*, gastroesophageal reflux disease; *NAFLD*, non-alcoholic fatty liver disease

**Table 2:** Perioperative outcomes of patients with deep staple line leak after SG

Outcome	
Concomitant surgery, *n* (%)	7 (11.4%)
Intraoperative complications, *n* (%)	12 (19.7%)
CD group	
CD 1–2, *n* (%)	37 (60.7%)
CD 3–5, *n* (%)	24 (39.3%)
Hospital length of stay (days), mean (SD)	22.3 (20.2)
ICU admission, *n* (%)	5 (8.2%)
Reoperations, *n* (%)	17 (27.8%)
> 2 reinterventions	16 (26%)
Readmission, *n* (%)	36 (59%)
Perioperative mortality, *n* (%)	2 (3.3%)

*SG*, sleeve gastrectomy; *CD*, Clavien-Dindo; *ICU*, intensive care unit

**Fig. 1:**
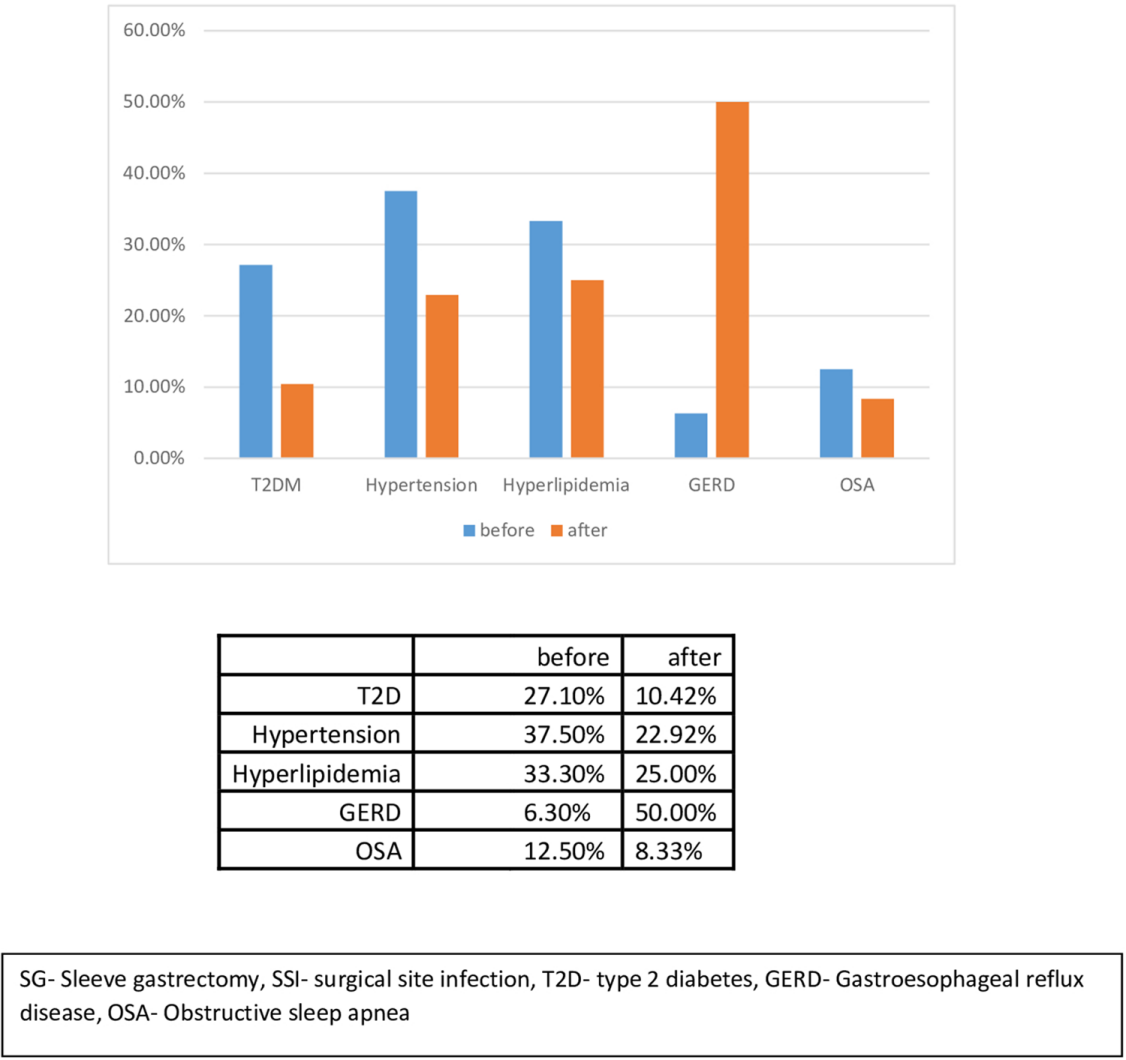
Rates of pre- and post-SG obesity associated medical problems in patients with staple line leak

Long-term follow-up was available in 78.7% of the study cohort (*n* = 48) and is shown in Table 3. Median follow-up time was 121 months, with median TWL of 27% ± 11.9 and BMI of 30.1 ± 4.9 kg/m^2^. The rate of chronic complications was 43.7% (*n* = 21), and 14.6% (*n* = 7) required revisional surgery during the follow-up. During the follow-up, 7 patients (14.6%) had symptomatic strictural disease of the sleeve, three of them were resolved after endoscopic dilation and the other four patients required revisional surgery—one completion to total gastrectomy and three patients were converted to Roux-en-Y gastric bypass (RYGB). Two patients (4.2%) developed a fistular disease during the follow-up, one was treated endoscopically with resolution and the other required a completion to total gastrectomy. Two patients were converted to one anastomosis gastric bypass (OAGB) due to insufficient weight loss. We also enocuntered patients with micronutrient deficency during the follow-up with iron deficiencey being the most common (*n* = 10, 20.8%), followed by vitamin B12 deficiency (*n* = 8, 19.5%), vitamin D deficiency (*n* = 4, 8.2%), and folate deficiencey (*n* = 3, 6.2%). Long-term effect on obesity-associated medical problems is shown in Table 4 and Fig. 1—all have improved compared to the preoperative status, except for GERD which increased significantly from 6.6% preoperatively to 50% in last follow-up. All patients (*n* = 4) who had a preoperative diagnosis of GERD did not have resolution of symptoms and 20 of the remaining 44 patients (45%) developed GERD during follow-up.

**Table 3:** Long-term follow-up of patients with staple line leak after SG

		*n*	(%)
Time of follow-up (months), mean (SD)	121 (19.2)		
BMI, mean (SD)	30.15 (4.9)		
%TWL, mean (SD)	27.31 (11.9)		
Chronic structural disease		7	14.58%
Fistular disease		2	4.1%
Reoperation		7	14.58%
Iron deficiency		10	20.8%
Vitamin D deficiency		4	8.2%
Vitamin B12 deficiency		8	19.5%
Folate deficiency		3	6.2%

*SG*, sleeve gastrectomy; *BMI*, body mass index; *SD*, standard deviation; *TWL*, total weight loss

**Table 4:** Rates of pre- and post-SG obesity associated medical problems in patients with staple line leak

	Baseline	Resolution	De novo
T2D % (*n*)	27.1% (13)	61.5% (8)	0% (0)
Hypertension % (*n*)	37.5% (18)	38.9% (7)	0% (0)
Hyperlipidemia % (*n*)	33.3% (16)	68.75% (11)	14.6% (7)
GERD % (*n*)	6.3% (3)	0% (0)	43.8% (21)
OSA % (*n*)	12.5% (6)	66.7% (4)	4.2% (2)

*SG*, sleeve gastrectomy; *T2D*, type 2 diabetes; *GERD*, gastroesophageal reflux disease; *OSA*, obstructive sleep apnea

In a comparative analysis of long-term outcomes to the control group (without staple line leak) with a median follow-up of 115 months, the mean BMI was meaningfully lower (30.15 ± 4.9 vs 36.3 ± 9.9; *p* ≤ 0.001) and the mean TWL was signifcantly higher in the control group (27.31% ± 11.9 vs 19.11% ± 16.8; *p* = 0.005). The reoperation rate was not meaningfully different at last follow-up (14.6% vs 22.0%; *p* = 0.278).

## Discussion

Staple line leak is an uncommon yet profound complication of SG. We believe those patients with organ space infection abutting the staple line without overt leak experienced a micro-leak as a port of entry for the infectious complication. In our study, staple line leak occurred in 3.1% of patients undergoing SG. Intraoperative complications, most commonly bleeding, occurred in 19.7% of patients with leak. About half of patients with staple line leak were managed conservatively (49.8%) and about half (50.8%) required invasive intervention and a quarter of these patients required 2 or more interventions. The perioperative mortality of patients with leak was 3.3%. Patients with staple line leak experienced a long post-operative hospitalization of 22 days on average. Long-term outcomes of those patients with deep SSI following SG were acceptable with meaningful %TWL and resolution of obesity-associated medical problems seen in these patients. After median follow-up of 121 months, median %TWL was 27%, and resolution of T2D, hypertension, hyperlipidemia, and obstructive sleep apnea was seen in 72%, 59%, 37%, and 56%, respectively. Revisional surgery was required in 14.6% of patients during follow-up. GERD was found in 50% of patients on long-term follow-up.

Staple line leak is a challenging complication after SG, occurring in 0.7 to 5% of patients [13]. Leaks usually occur in the proximal part of the staple line near the angle of His. The causes for leak at this area are likely to be due to relative ischemia and increased wall tension. The arterial supply of the gastric sleeve is based on the right and left gastric arteries. The proximal staple line is far away from this blood supply and is relatively avascular following SG transection of the stomach, since it is usually supplied by the ligated short gastric vessels. Also contributing to relative tissue ischemia at this point is increased wall tension. This proximal part of gastric sleeve is usually also the widest part, and since wall tension is proportionate to the diameter (according to Le Places’ law), wall tension is greatest at this area, hindering micro-capillary blood flow. In fact some have described lower leak rate when using large bougies (≥ 40F), perhaps due to more even distribution of wall tension along the gastric sleeve [14].

SG is efficacious in treating obesity and its associated comorbidities. In a systematic review of studies with at least 10-year follow-up [15], the mean TWL was 24.4% (17–36.9%), T2D remission was 45.6%, and HTN remission was 41.4%. The prevalence of de novo GERD was 32.3%. Revisional surgery was required in 19.2% of patients with RYGB being the most common conversion. Long-term results of patients experiencing staple line leak following SG in our study are not meaningfully different than those described in this systematic review, suggesting that those patients with staple line leak who survive the index admission do not have worse long-term outcomes to those who do not have this complication. In fact, we showed that when comparing the study group to a control group without staple line leaks, these patients showed higher TWL and lower BMI. However, it is also possible that this may be a result in part due to poor intake and catabolism caused by the infectious complications in these patients and not due to the efficacy of the SG procedure alone. The comparative groups may be too small to draw clear conclusions from this analysis.

Management of leak following SG depends on the day of onset and the clinical severity of the leak [16]. Early leaks (POD1-4) and leaks causing hemodynamic instability and/or end organ damage are usually treated operatively [17]. Previous series have shown re-operation and re-intervention rates of 30–75% and 88–100% with 63–80% of patients requiring multiple procedures [16, 18, 19]. In contrast, in our cohort, the re-operation rate and re-intervention rate were 27.8% and 50.8%, respectively, with 26% of patients requiring more than one intervention. This may be due to inclusion of patients without overt leaks. Benedix and colleagues [18] investigated a prospective country-wide registry in Germany and compared 241 patients with leak following SG with 15,756 who did not experience leak. They found a 9% intra-operative complication rate, a 3.7% perioperative mortality rate and a median LOS of 22 days for patients with leak, all of which are similar to our findings (19.7%, 3.3%, and 22 days, respectively). In contrast to our findings, reoperation was performed in 75% of patients in their study in comparison to 27.8% in our cohort. In line with our findings, Benedix and colleagues described that in a follow-up of 24 months outcomes including weight loss and resolution of comorbidities was not meaningfully different between patients that experienced leak and those that didn't at 12 and 24 months following surgery [15]. This study showed lower prevalence of GERD on long-term follow-up than in our study (22% vs 50% respectively) and the authors did not find a meaningful difference in the prevalence of GERD between patients with and without leak. The higher prevalence of GERD in our study may be due, in part, to the longer follow-up of ten rather than 2 years. In a retrospective single-center study from France, Rebibo and colleagues [19] investigated weight loss parameters and resolution of comorbidities between patients that experienced leak to those that did not, by 1:2 propensity score matching at 12-month follow-up; similar to our findings and those of Benedix and colleagues, no difference was found. In line with our cohort, Rebibo and colleagues described a perioperative mortality rate of 4.1% and mean LOS of 30 days. Similar to Benedix and colleagues, and in contrast to our findings, Rebibo and colleagues described a reoperation rate of 73%.

The limitations of our study include potential selection bias and under-reporting inherent in the retrospective design, the small number of patients, and lack of generalizability due to being conducted at a single center. Additionally, long-term follow-up information was only available for 48/61 patients, leading to additional possibility of bias. The strengths of this study include the length of follow-up of more than 10 years on average, and the granularity of the clinical data. A prospective study with a multicenter involvement could yield more substance to the already existing literature.

## Conclusions

In conclusion, deep SSI occur in around 3% of patients undergoing SG and are associated with a 3% mortality rate. In this study, satisfactory perioperative outcomes were achieved in a majority of patients with non-operative management and in a large minority of patients with non-interventional management. It is our opinion that deep SSI without overt leak still arise from micro-leak as a port of entry. Although staple line leak is associated with intra-operative complications and a relatively long LOS, the long-term outcomes for these patients in terms of weight loss and resolution of comorbidities is not worse than those reported for patients that did not staple line leak.

### Supplementary Information

Below is the link to the electronic supplementary material.

Supplementary file1 (DOCX 266 KB)

Supplementary file2 (DOCX 263 KB)

## Data Availability

The datasets generated during and/or analysed during the current study are available from the corresponding author on reasonable request.
