# Supplementary material for: Long-Term Outcomes of Patients with Staple Line Leaks Following Sleeve Gastrectomy
Source: Obes Surg. 2024 May 30;34(7):2523–9. doi: 10.1007/s11695-024-07307-0 (PMC11217129; doi:10.1007/s11695-024-07307-0)
Supplement: Supplementary file 1 — Supplementary file1 (DOCX 266 KB) [file 11695_2024_7307_MOESM1_ESM.docx]

| **Characteristic** | **Study Cohort (n=61)** | **Control Group (n=77)** | **P value** |  |
| --- | --- | --- | --- | --- |
| **Age, mean (SD)** | 39.8 (11.3) | 40.8 (13.9) | 0.640 |  |
| **Male gender, n (%)** | 25 (41.0%) | 23 (29.9%) | 0.173 |  |
| **BMI, mean (SD)** | 41.5 (4.7) | 43.2 (4.7) | 0.150 |  |
| **T2D, n (%)** | 18 (29.5%) | 19 (24.7%) | 0.524 |  |
| **Hypertension, n (%)** | 27 (44.3%) | 31 (40.2%) | 0.639 |  |
| **Hyperlipidemia, n (%)** | 19 (31.2%) | 29 (37.7%) | 0.425 |  |
| **GERD, n (%)** | 5 (8.2%) | 12 (15.6%) | 0.192 |  |

**Supplementary Table 1 – Comparison of baseline characteristics of patients with staple line leak after SG to patients without staple line leak.**

**SG- Sleeve Gastrectomy, SD- Standard deviation, BMI- Body mass index, T2D- Type 2 diabetes, GERD- Gastroesophageal reflux disease**
