# Supplementary material for: Long-Term Outcomes of Patients with Staple Line Leaks Following Sleeve Gastrectomy
Source: Obes Surg. 2024 May 30;34(7):2523–9. doi: 10.1007/s11695-024-07307-0 (PMC11217129; doi:10.1007/s11695-024-07307-0)
Supplement: Supplementary file 2 — Supplementary file2 (DOCX 263 KB) [file 11695_2024_7307_MOESM2_ESM.docx]

**Supplementary Table 2 – Comparison of long-term follow-up of patients with staple line leak after SG to patients without staple line leak.**

|  |  |  |  |  |  |
| --- | --- | --- | --- | --- | --- |
|  | | **Study Cohort** | **Control Group** | **P value** |  |
| **BMI (Kg/m^2^) , mean (SD)** | | 30.15 (4.9) | 36.30 (9.9) | **<0.001** |  |
| **%TWL, mean (SD)** | | 27.31 (11.9) | 19.11 (16.8) | **0.005** |  |
| **Reoperation N (%)** | | 7 (14.6%) | 17 (22.0%) | 0.278 |  |

SD – Standard deviation, SG- Sleeve Gastrectomy, BMI- Body mass index, TWL- total weight loss
